# Development and external validation of a nomogram for neurosyphilis diagnosis among non-HIV patients: a cross-sectional study

**DOI:** 10.1186/s12883-021-02454-8

**Published:** 2021-11-18

**Authors:** Wenjing Ge, Yang Zhang, Chao Peng, Dongdong Li, Lijie Gao, Jiajia Bao, Changling Li, Ning Chen, Dong Zhou, Li He

**Affiliations:** 1 Department of Neurology, West China Hospital, Sichuan University, Chengdu, People’s Republic of China; 2 Department of Laboratory Medicine, West China Hospital, Sichuan University, Chengdu, People’s Republic of China

**Keywords:** Neurosyphilis, Diagnostic model, HIV-negative patient, Serum TRUST, Psychiatric behaviour disorders

## Abstract

**Background:**

The diagnosis of neurosyphilis is challenging due to the requirement of a lumbar puncture and cerebrospinal fluid (CSF) laboratory tests. Therefore, a convenient diagnostic nomogram for neurosyphilis is warranted. This study aimed to construct diagnostic models for diagnosing neurosyphilis.

**Methods:**

This cross-sectional study included data of two patient cohorts from Western China Hospital of Sichuan University between September 2015 and April 2021 and Shangjin Hospital between September 2019 and April 2021 as the development cohort and the external validation cohort, respectively. A diagnostic model using logistic regression analysis was constructed to readily provide the probability of diagnosis at point of care and presented as a nomogram. The clinical usefulness of the diagnostic models was assessed using a receiver operating characteristic (ROC) and Harrell concordance (Harrell C) index for discrimination and calibration plots for accuracy, which adopted bootstrap resampling 500 times.

**Results:**

One hundred forty-eight and 67 patients were included in the development and validation cohorts, respectively. Of those, 131 were diagnosed as having reactive neurosyphilis under the criteria of positive results in both CSF treponemal and non-treponemal tests. In the development cohort, male, psychiatric behaviour disorders, and serum toluidine red unheated serum test were selected as diagnostic indicators applying a stepwise procedure in multivariable logistic model. The model reached 80% specificity, 79% sensitivity, and 0·85 area under the curves (AUC) (95% confidence interval, 0·76–0·91). In the validation cohorts, the Harrell C index for the diagnostic possibility of reactive neurosyphilis was 0·71.

**Conclusions:**

A convenient model using gender, presence of psychiatric behaviour disorders, and serum TRUST titre was developed and validated to indicate diagnostic results in patients suspected of neurosyphilis. Checking the model value of factors on nomogram is a feasible way to assist clinicians and primary health servers in updating patients’ medical charts and making a quantitatively informed decision on neurosyphilis diagnosis.

**Trial registration:**

This research was retrospectively registered in the Ethics committee on biomedical research, West China Hospital of Sichuan University. The research registration and committee’s reference number was 1163 in 2020 approval.

**Supplementary Information:**

The online version contains supplementary material available at 10.1186/s12883-021-02454-8.

## Background

Neurosyphilis (NS) is one of the most feared complications of syphilis [1], and dissemination of the pathogenic bacterium of neurosyphilis, *Treponema pallidum* subspecies *pallidum*, to the cerebrospinal fluid (CSF) and meninges can occur at any stage of the infection [1]. Importantly, the injury to brain tissues caused by *Treponema pallidum* invasion is irreversible [1]. Trend results from syphilis notification data in the 25 countries with comprehensive surveillance systems showed an increase, especially in Europe, of up to 70% since 2000 [2, 3]. However, the proportion of neurosyphilis among patients with syphilis is undetermined due to diagnostic limitations and requirement of skilful doctors to perform lumbar puncture and lab operators for special tests [4]. Further, necessary resources are usually not available in primary community healthcare centres of urban districts or common hospitals in smaller areas [5].

Prior to the advent of antibiotics, the typical symptoms of neurosyphilis, such as Argyll Robertson pupils, were used to diagnose neurosyphilis [1]. However, access to antibiotics has greatly increased and affected the disease process and manifestation of neurosyphilis [6]. Whether only signs and symptoms can be used for neurosyphilis identification is controversial. In the recent years, headache and blurred vision were reported as supportive factors for neurosyphilis diagnosis [7, 8], while other reports suggested various clinical manifestations of neurosyphilis with a lack of specificity [9]. Furthermore, most of the descriptions of neurosyphilis symptoms are derived from reports on American cohorts co-infected with HIV [8, 10] and there is a lack information on non-HIV patients with neurosyphilis, who constitute the majority of patients with neurosyphilis in Europe and Asia [9, 11].

The laboratory diagnosis of neurosyphilis was putatively based on positive results from serum and CSF serologic tests, as well as elevations in the CSF white cell count and protein levels [12, 13]. In 2015, an American guideline from the Centers for Disease Control and Prevention (CDC), U.S. Department of Health and Human Services, suggested the use of a decision tree for neurosyphilis diagnosis, which required a positive or reactive definition of non-specific, specific, or alternative tests in CSF for patients suspected with neurosyphilis [12]. A European guideline of the European Academy of Dermatology and Venerology recommends the cut-off value of CSF treponemal tests in patients co-infected with HIV [13]. Recent studies estimating serologic cut-off values have found that the accuracy of neurosyphilis diagnosis depends on the choice of controls with various clinical characteristics [14]. Combined usage of diagnostic tests for neurosyphilis needs further validation in the post-antibiotics era.

Variables in continuous form, instead of the traditional binary form as positive or negative report results referring to a certain threshold, can be utilised completely in the current analysis strategies for evidence-based medicine [15]. By combining continuous variables with clinical parameters and presenting them as a visual graph, a nomogram makes the results of a diagnostic model simpler to use. Furthermore, this single numerical estimate of the probability of an event facilitates the evaluation of patients with neurosyphilis, especially in poor areas that lack expert operators and resources to perform time-consuming tests [16]. Serum tests and CSF assessments have been extensively discussed as objective indicators of neurosyphilis, supporting the diagnosis; however, their usefulness remains inconclusive [8, 17]. The present study was designed to verify and explore the association between these factors for diagnostic confirmation using retrospective patient data. Using the patient clinical and laboratory characteristics, we developed a feasible diagnostic nomogram to assess the possibility of neurosyphilis in an HIV-negative population with an unknown syphilis duration, and further validated its validity and the score obtained.

## Methods

This study included consecutive patients who presented with positive results of serum treponemal (*Treponema pallidum* chemiluminescence assay [TP CLIA] or *Treponema pallidum* particle agglutination assay [TPPA]) and a non-treponemal serological test (toluidine red unheated serum test [TRUST]) at the West China Hospital, Medical College of Sichuan University, from September 2015 to April 2021 and at Shangjin Hospital between September 2019 and April 2021. Patients met one of the following criteria: presence of neurological or ophthalmological symptoms or signs (such as headache, photophobia, blurred version, confusion, sleep disorders, vertigo, hearing loss, version loss, confusion, lethargy, memory change, progressive dementia, psychiatric behaviour disorders, personality change, numbness, fatigue or pain in limbs and trunk, seizure, tremor) and no symptom or syphilis of unknown duration, or failure of antibiotic treatment (titre of serum non-treponemal test failing to decrease by 4-folds or unable to serorevert following antibiotic treatment).

All methods were carried out in accordance with the Transparent Reporting of a multivariable prediction model for Individual Prognosis or Diagnosis (TRIPOD) guidelines and regulations.

The ethics committee on biomedical research, West China Hospital of Sichuan University approved the study and waived the need for informed consent from all subjects. The committee’s reference number was 1163 in 2020 approval.

### Diagnostic criteria

**Fig. 1:**
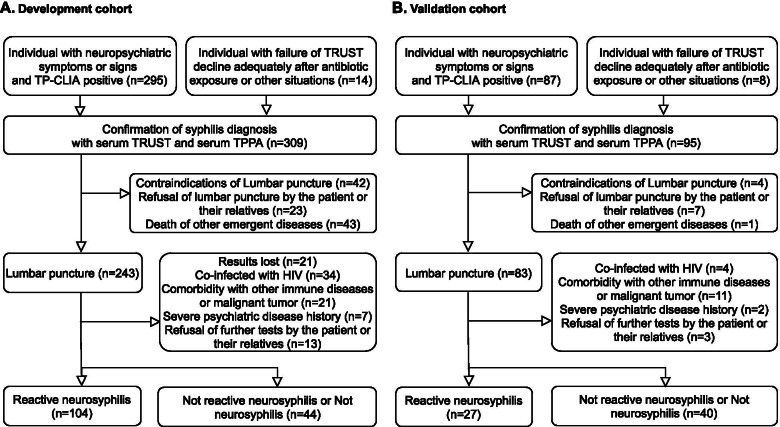
Participant flow diagram. Individuals with neuropsychiatric symptoms or signs and those who were TP-CLIA positive, individuals with failure of TRUST decline after antibiotic exposure, and others (with TP-CLIA positive) who requested further diagnosis were included (**A**). Reactive neurosyphilis refers to syphilis patients with positive results in both CSF-TRUST and CSF-TPPA. Non-reactive neurosyphilis refers to syphilis patients with negative results in CSF-TRUST but positive results in CSF-TPPA. Syphilis patients with negative results in both CSF-TRUST and CSF-TPPA were regarded as “Not neurosyphilis” (**B**). CSF, cerebrospinal fluid; TRUST, Toluidine red unheated serum test; TP-CLIA, *Treponema pallidum* chemiluminescence assay; TPPA, *Treponema pallidum* particle agglutination assay

Subjects who were enrolled at West Hospital of Sichuan University (*n* = 309) formed the development cohort and those enrolled at Shangjin Hospital (*n* = 95) formed the validation cohort (Fig. 1). The diagnostic criteria for neurosyphilis were based on the guidelines of the CDC in the USA and Europe and related literature [10, 11]. We applied a strict diagnostic criterion with a combination of two CSF laboratory methods to ensure diagnostic specificity among the suspected participants. Patients with double positive results in both the CSF TRUST and CSF TPPA tests were assigned to the confirmed reactive neurosyphilis group, and the others were assigned to the control group.

### Laboratory methods

Serum samples were collected within 4 days of the lumbar puncture [8]. A serum non-treponemal test, TRUST (Rongsheng, Shanghai, China) and treponemal test, TPPA (Fujirebio, Tokyo, Japan) or chemiluminescent immunoassay (CLIA; Fujirebio, Tokyo, Japan) and Lumipulse G TP-N syphilis (Fujirebio, Tokyo, Japan) were performed.

### Statistical analysis

Associations between categorical variables were assessed using a chi-squared test or Fisher’s exact test. Associations between continuous variables and categorical variables were assessed using the Mann–Whitney U test. Diagnostic factors were analysed and selected using univariable and multivariable regression analyses in a stepwise manner, for confirmation of reactive neurosyphilis and the other group including non-reactive and not neurosyphilis – as a binary classification out of clinical consideration. A two-tailed *P* value > 0·05 was used for the removal of variables. CSF items from clinical guidelines were assessed for possible additional effects, among which the collinearity was tested. Boot strapping was resampled 500 times to obtain a 95% confidence interval (CI) and quantify the effects of diagnostic indicator selection strategies on the model development. Performance measures included the average area under the ROC curve, sensitivity, specificity, a calibration plot, and the Harrell concordance (Harrell C) index. All analyses were weighted according to the analytical guidelines. *P*-values < 0·05 were considered statistically significant. R software (version 3.3.1; http://www.R-project.org) was used for the analysis.

## Results

### Demographic and clinical features

The demographic and clinical features of the 215 clinically suspected patients with neurosyphilis with complete information of treponemal and non-treponemal serum and CSF examination are summarized in Table 1. Among the 148 patients in the development cohort, 83·7% were male; 14 (21·3%) were non-symptomatic patients and required a CSF test. Further, 105 patients (70·9%) did not receive any prior antibiotic treatment. The most common symptoms of neurosyphilis were psychiatric behaviour disorders (50·0% in the development and 25·9% in the validation cohort). Other symptoms such as sleep disorders, photophobia, and blurred version were also observed (Supplement 1).

**Table 1:** Demographic and clinical features of the participants

Diagnosis	Development Cohort			Validation Cohort		
	Not or non-reactive NS	Reactive NS	*P* value	Not or non-reactive NS	Reactive NS	*P* value
N	44	104		40	27	
Sex, n (%)			0·219			0·432
Male	33 (75·0)	87 (83·7)		26 (65·0)	20 (74·1)	
Female	11 (25·0)	17 (16·3)		14 (35·0)	7 (25·9)	
Age, years, mean (SD)	46·4 (13·7)	47·3 (11·8)	0·694	48·75 (13·40)	52·96 (16·32)	0·252
≥45	22 (50·0)	62 (59·6)	0·19			
< 45	22 (50·0)	42 (40·4)				
Height, cm, mean (SD)	164·3 (7·8)	166·5 (7·7)	0·208	165·50 (8·46)	166·63 (7·98)	0·585
Weight, kg, mean (SD)	61·4 (9·6)	62·9 (11·4)	0·542	71·13 (20·89)	68·92 (12·59)	0·642
Education level, n (%)			0·110			0·197
High school or beyond	19 (44·2)	30 (30·3)		13 (32·5)	13 (48·1)	
Beyond high school	24 (55·8)	69 (69·7)		27 (67·5)	14 (51·9)	
Address, n (%)			0·088			0·202
City	24 (55·8)	33 (33·0)		9 (22·5)	5 (18·5)	
Urban-rural fringe area	5 (11·6)	14 (14·0)		6 (15·0)	5 (18·5)	
Village and town	14 (32·6)	47 (47·0)		18 (45·0)	17 (63·0)	
Other province of China	0 (0·0)	4 (4·0)		6 (15·0)	0 (0·0)	
Aboard	0 (0·0)	2 (2·0)		1 (2·5)	0 (0·0)	
Treatment before, n (%)	15 (43·9)	28 (27·7)	0·207	22 (55·0)	12 (44·4)	0·347
Clinical symptoms, n (%)						
No symptom	6 (13·6)	8 (7·7)	0·259	5 (12·5)	3 (11·1)	0·863
Psychiatric behaviour disorders	9 (20·5)	52 (50·0)	< 0·001	8 (20·0)	7 (25·9)	0·568
Memory change	9 (20·5)	40 (38·5)	0·033	7 (17·5)	5 (18·5)	0·915
Sleep difficulty	5 (11·4)	23 (22·1)	0·127	1 (2·5)	1 (3·7)	0·776
Photophobia	6 (13·6)	8 (7·7)	0·259	2 (5·0)	1 (3·7)	0·801
Blurred Version	4 (9·1)	6 (5·8)	0·462	3 (7·5)	1 (3·7)	0·520
TP CLIA, mean (SD)	–	–	–	57·3 (42·0)	93·3 (49·9)	0·002
1 < X < 100, n (%)	7 (16·3)	1 (1·0)		24 (60·0)	7 (25·9)	
X > 100, n (%)	36 (83·7)	99 (99·0)		16 (40·0)	20 (74·1)	
Creatine kinase, mean (SD), U/L	106·5 (137·3)	196·5 (373·0)	0·132	–	63·3 (15·1)	–
Elevated CK, n (%)	2 (4·8)	20 (20·6)	0·019	–	0 (0)	–
Serum TRUST, n (%)			< 0·001			0·008
Negative	9 (22·5)	3 (3·0)		15 (37·5)	1 (4·0)	
1	14 (35·0)	9 (9·0)		19 (47·5)	11 (44·0)	
8	5 (12·5)	7 (7·0)		4 (10·0)	5 (20·0)	
16	6 (15·0)	18 (18·0)		1 (2·5)	3 (12·0)	
32	4 (10·0)	30 (30·0)		0 (0·0)	1 (4·0)	
≥ 64	2 (5·0)	33(33·0)		1 (2·5)	4 (16·0)	
Serum TPPA > 1:320, n (%)	36 (81·8)	96 (92·3)	0·060	35 (87·5)	25 (96·2)	0·130

*Note*: *SD* Standard deviation, *Q* Quartile; Neurosyphilis, NS; Elevated creatine kinase (CK) male ≥308, female ≥192 U/L; *TP CLIA Treponema pallidum* chemiluminescence assay; TRUST, Toluidine red unheated serum test, *TPPA Treponema pallidum* particle agglutination assay, IgG Immunoglobulin. Data with normal distribution are described using mean (SD)

### Laboratory findings and diagnostic yield

The univariable logistic regression analysis revealed that male sex and psychiatric behaviour disorders had a higher likelihood of a reactive neurosyphilis diagnosis, with odds ratio (OR) of 1·71 (95% CI 0·72–4·02, *P* = 0·222) and 3·89 (95% CI 1·70–8·89, *P* = 0·0001) respectively. Compared to the control group, the titre of serum TRUST above 1:16 had a significantly higher likelihood of a reactive neurosyphilis diagnosis, with (OR 9·00, 95% CI 1·82–44·59, *P* = 0·007). When creatine kinase was treated as a binary variable, elevated creatine kinase showed an association with reactive neurosyphilis (OR, 4·8; 95% CI, 1·1–21·8, *P* = 0·042, Table 2).

**Table 2:** Univariable logistic regression analysis of each indicator and diagnostic in the development cohort

Characteristic	Univariable analysis		
	Odds ratio	95% CI	*P* value
Male	1·71	(0·72, 4·02)	0·222
Age	1·01	(0·98, 1·04)	0·691
Treatment before	0·53	(0·25, 1·11)	0·094
Psychiatric behaviour disorders	3·89	(1·70, 8·89)	0·001
No symptom	0·53	(0·17, 1·62)	0·263
Serum TPPA > 1:320	2·67	(0·93, 7·64)	0·068
Serum TRUST			
Negative	Ref		
1	1·93	(0·41, 9·10)	0·407
8	4·20	(0·74, 23·91)	0·106
16	9·00	(1·82, 44·59)	0·007
32	22·50	(4·23, 119·77)	0·000
64	49·50	(7·15, 342·77)	< 0·0001
Serum creatine kinase	1·00	(1·00, 1·01)	0·109
Elevated CK	4·8	(1·1, 21·8)	0·042
Serum IgG	1·03	(0·90, 1·18)	0·687
Serum albumin	0·95	(0·88, 1·04)	0·284
CSF Protein	6·51	(1·77, 23·96)	0·005
≤ 0·45	Ref		
> 0·45	4·80	(2·22, 10·36)	< 0·0001
CSF Glucose	0·66	(0·39, 1·09)	0·105
CSF Nucleated cells	1·00	(0·99, 1·01)	0·899
≤ 5	Ref		
> 5	2·89	(1·38, 6·08)	0·005

*TPPA Treponema pallidum* particle agglutination assay, *TRUST*, Toluidine red unheated serum test; Elevated creatine kinase (CK) male ≥308, female ≥192 U/L, *CSF* Cerebrospinal fluid, *IgG* Immunoglobulin

Multivariable logistic analysis shown in Table 3 indicated that the following factors were more likely related to reactive neurosyphilis: serum TRUST 1:8 (OR, 7·9; 95% CI, 1·1–54·5), 1:16 (OR, 16·0; 95% CI 0·6–97·7), 1:32 (OR, 33·6; 95% CI, 5·4–209·7), and 1:64 (OR, 71·9; 95% CI, 9·1–570·0), psychiatric behaviour disorders (OR, 5·1; 95% CI, 1·7–15·4), and male (OR, 1·00; 95% CI, 0·3–3 ·1).

**Table 3:** Multivariable logistic analysis for the construction of diagnostic models

	Development cohort		Validation cohort	
	Multivariable	*P*	Multivariable	*P*
Male	1·0 (0·3, 3·1)	0·954	1·5 (0·5, 4·5)	0·43
Psychiatric behaviour disorders	5·1 (1·7, 15·4)	0·004	1·4 (0·4, 4·5)	0·57
Serum TRUST				
Negative	Ref		Ref	
1	3·2 (0·6, 18·8)	0·194	8·7 (1·0, 75·0)	0·05
8	7·9 (1·1, 54·5)	0·036	18·8 (1·7, 209·6)	0·02
16	16·0 (2·6, 97·7)	0·003	45·0 (2·2, 937·4)	0·01
32	33·6 (5·4, 209·7)	< 0·001	inf· (0·0, Inf)	0·99
64	71·9 (9·1, 570·0)	< 0·001	60·0 (3·0, 1185.1)	0·01

*TRUST* Toluidine red unheated serum test, *IgG* Immunoglobulin, *CSF* Cerebrospinal fluid

The sensitivity and specificity of the diagnostic model in the development cohort were 80·0 and 79·0 respectively, and those in the validation cohort were 77·5 and 81·5, respectively (Table 4). The positive and negative perfective value was 90·8% and 60·3%, respectively. The receiver operating characteristics (ROC) curve of the model was similar between the development (0·85, 95% CI: 0·77–0·91) and validation cohorts (0·85, 95% CI: 0·74–0·93).

**Table 4:** Accuracy of the diagnostic score of the nomogram for estimating the risk of reactive neurosyphilis

Variable	Value	
	Development cohort	Validation cohort
ROC area (AUC*) 95% CI*	0·85 (0·77, 0·91)	0·85 (0·74, 0·93)
Specificity, %	80·0	77·5
Sensitivity, %	79·0	81·5
Positive predictive value, %	90·8	71·0
Negative predictive value, %	60·3	86·1

Receiver operating characteristics (ROC) curve of the model was generated using gender, psychiatric behaviour disorders, and serum TRUST for neurosyphilis diagnosis. Diagnose-odds ratio (OR), positive predictive value (pv), and negative pv are calculated. *Area under the curve (AUC), confidence interval (CI), and significance test adopted 500-time bootstrap resampling

**Fig. 2:**
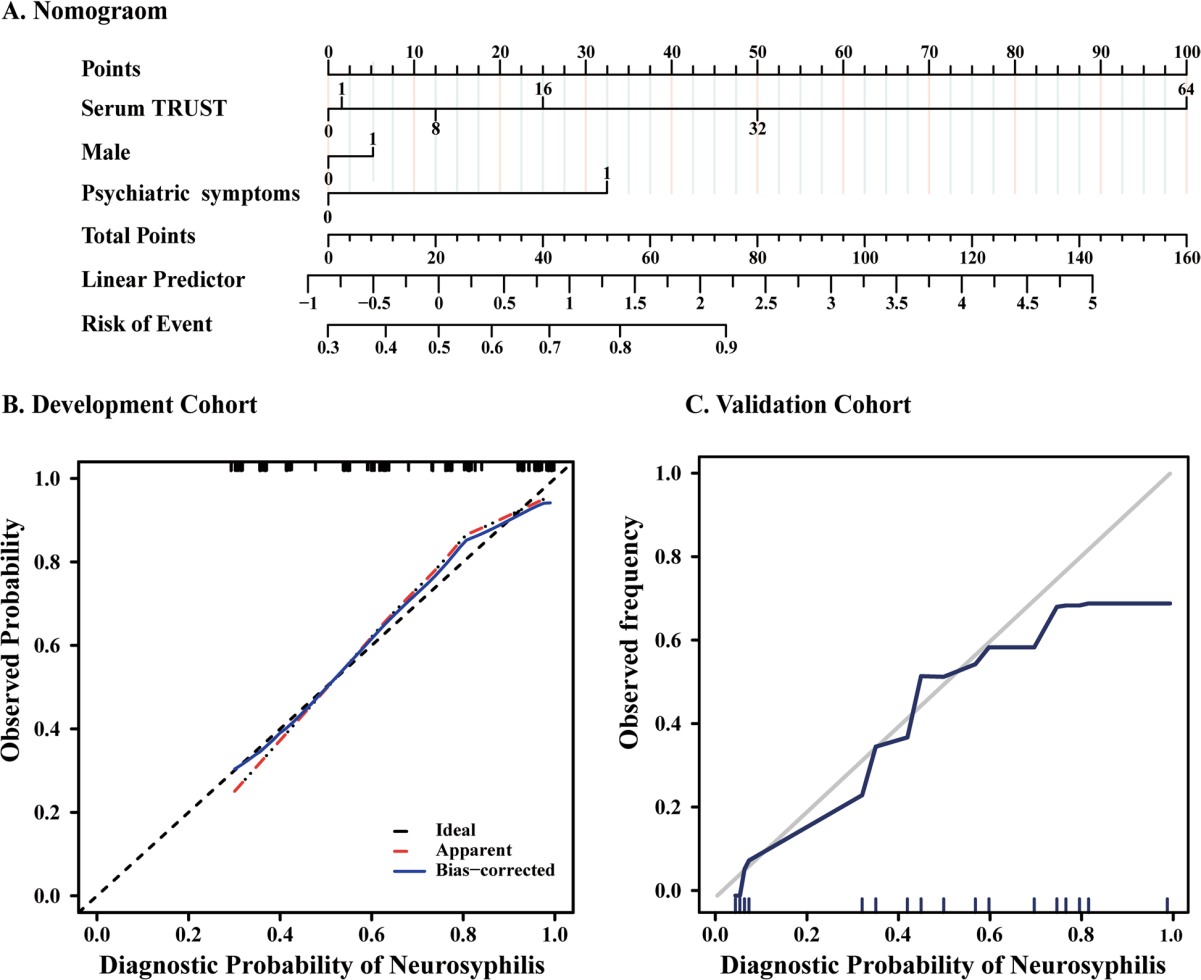
ROC curve, DCA, and nomograms. **A** Nomogram visualising the multivariable diagnostic model. To use the nomogram, find the position of each variable on the corresponding axis, draw a line to the points axis for the number of points, add the points from all variables, and draw a line from the total points axis to assess diagnostic probability of neurosyphilis at the lower line of the nomogram. **B** Calibration curves of the nomogram for the diagnostic probability of neurosyphilis in the training cohort (*n* = 148). **C** Validity of the performance of the nomogram in estimating the diagnostic probability of neurosyphilis in the validation cohort (*n* = 67). *AUC confidence interval and significance test adopt Bootstrap resampling 500 times

Figure 2 illustrates the nomogram with multi-variables for the diagnostic probability of reactive neurosyphilis (Fig. 2A). The nomogram demonstrated good accuracy in estimating the diagnostic probability of reactive neurosyphilis, with a bootstrap-corrected C index of 0·83 (Fig. 2B). The calibration plots were graphically good in the validation cohort; the nomogram displayed a C index of 0·71 for the estimation of reactive neurosyphilis diagnosis (Fig. 2C).

## Discussion

In this study, two important clinical findings were obtained. First, male sex, psychiatric behaviour disorders, and serum TRUST titre were useful indicators for the diagnosis of neurosyphilis without lumbar puncture. Second, a diagnostic model for neurosyphilis was developed and validated using clinical characteristics and laboratory test data of patients and transformed into a nomogram.

Binary form of Serum TRUST titres is commonly used, reporting positive or negative results in patients with syphilis. However, we found that exact serum the TRUST titre in conjugation with sex and psychiatric behaviour disorders could be used to differentiate patients with reactive neurosyphilis from those suspected of having neurosyphilis. Comparing the results with those for non-reactive neurosyphilis and not neurosyphilis patients, the serum TRUST levels were higher in those with reactive neurosyphilis. Cai et al. also indicated a 5-fold increased likelihood of asymptomatic neurosyphilis in patients with a serum TRUST titre ≥1:64 [17]. Researchers noted that an increase in the serum TPPA titre and serum creatine kinase could serve as a surrogate for CSF clinical abnormalities after lumbar punctures [17, 18]. Unfortunately, we were unable to determine the titre grades of serum TPPAs, since the laboratory system of our hospital automatically sets and reports serum TPPA titres > 1:320 as positive. Xiao et al. suggested that elevated serum creatine kinase was linked to the non-HIV neurosyphilis group (including probable neurosyphilis with negative CSF TRUST, elevated protein or white blood cells in cerebrospinal fluid) in comparison with not neurosyphilis, when precluding asymptomatic patients [18]. Our data for the development cohort showed that elevated serum creatine kinase also was linked to reactive neurosyphilis, when our research cohort included asymptomatic patients.

We used an exploratory approach combing clinical parameters and serum TRUST titres to develop diagnostic models for neurosyphilis. When combined with clinical parameters, the diagnostic performance was improved compared to that with the use of serum TRUST alone. The number of male patients was three times higher than that of female patients in our cohort, and similar to that reported by the Public Health England [19]. The rate of the presence of classic symptoms and photophobia found in this study was consistent with that reported by Arielle, and was approximately 10% in HIV-negative patients with neurosyphilis [9]. The rate of neurosyphilis typical symptoms like Argyll Robertson pupils was seldom reported. Instead, the significant clinical parameters observed in this study were psychiatric behaviour disorders and memory deterioration, consistent with the Canadian and European case series reports of neurosyphilis and research in North China from He et al. [20–23].

ROC curve analysis was used to assess the diagnostic model’s performance. Approximately 85% of the AUC of the diagnostic model was similar between the development and validation cohorts. When we consider that lumbar puncture may be difficult for patients, nomogram having about 80% diagnostic probability may be sufficient to suggest diagnosis of neurosyphilis. However, in most cases, an 80% threshold probability is not sufficient, especially in cases with a high threshold to perform continuous intravenous antibacterial therapy. In such cases, it is better to complete CSF tests and conduct more specific diagnostic tests, such as the Venereal Disease Research Laboratory (VDRL) on the CSF, a well-known specific test, broadly used to diagnose idiopathic neurosyphilis in America, although it is time-consuming and less feasible, especially in countries with high rates of such patients [24]. The results of this study offer a sensitive screening nomogram for advising candidates with high diagnostic possibility of reactive neurosyphilis to undergo lumbar puncture, complete CSF regular tests, or undergo CSF-VDRL measurements. Furthermore, this nomogram was cheaper than a regular procedure in terms of prices of medical services and tests, referring to prices of these items in 2021 from West China hospital (Supplement 2).

This study had several limitations. First, it might have a sampling bias. We did not exclude patients who received insufficient antibiotic therapy before lumbar puncture. Under the current criterion of group assignment, false negatives were possible due to non-reactive neurosyphilis cases. In theory, disease duration should have been analysed as a risk factor for neurosyphilis, but it was difficult for patients with neuropsychological symptoms to provide the exact time of syphilis infection or information on sexual activities. Additionally, another limitation was the small sample size and that the pathological categories of neurosyphilis with sophisticated infectious degree and loci were not employed here. Our data (nine records of creatine kinase in validation cohort) were not sufficient to validate a model with the addition of an elevated creatine kinase level. Whether patients in each dedicated category had a different prognosis remains unknown due to lack of follow-up investigation. We intend to build a systematic database and prospectively design new studies to improve the quality of the evidence and facilitate more comprehensive patient care. In conclusion, to verify the validity of this model, future studies are warranted.

## Conclusion

Importantly, an economical nomogram can be offered to assist clinicians and primary health servers in updating patient medical charts and making informed decisions on neurosyphilis diagnosis.

## Supplementary Information

**Additional file 1: Supplement 1** Clinical symptoms and comorbidities. **Supplement 2** Cost of neurosyphilis diagnosis tests.

## Data Availability

The datasets used and/or analysed during the current study available from the corresponding author on reasonable request.

## Abbreviations

CSF: Cerebrospinal fluid
NS: Neurosyphilis
TP: *Treponema pallidum*
TPCA: *Treponema pallidum* chemiluminescence assay
TPPA: *Treponema pallidum* particle agglutination assay
TRUST: Toluidine red unheated serum test
VDRL: Venereal Disease Research Laboratory
