# Supplementary material for: Development and external validation of a nomogram for neurosyphilis diagnosis among non-HIV patients: a cross-sectional study
Source: BMC Neurol. 2021 Nov 18;21:451. doi: 10.1186/s12883-021-02454-8 (PMC8600785; doi:10.1186/s12883-021-02454-8)
Supplement: Supplementary file 1 — Additional file 1: Supplement 1 Clinical symptoms and comorbidities. Supplement 2 Cost of neurosyphilis diagnosis tests. [file 12883_2021_2454_MOESM1_ESM.docx]

**Supplement 1 Clinical symptoms and comorbidities**

| Diagnosis | Development Cohort | | | Validation Cohort | | |
| --- | --- | --- | --- | --- | --- | --- |
|  | Not or non-reactive NS | Reactive NS | P value | Not or non-reactive NS | Reactive NS | P value |
| N | 44 | 104 |  | 40 | 27 |  |
| **Symptom** |  |  |  |  |  |  |
| Headache | 4 (9·09) | 11 (10·58) | 0·784 | 6 (15·0) | 2 (7·4) | 0·347 |
| Psychiatric behaviour disorders | 9 (20·45) | 52 (50·00) | <0·001 | 8 (20·0) | 7 (25·9) | 0·568 |
| Memory change | 9 (20·45) | 40 (38·46) | 0·033 | 7 (17·5) | 5 (18·5) | 0·915 |
| Seizure | 1 (2·27) | 12 (11·54) | 0·069 | 0 (0·0) | 0 (0·0) | - |
| Sleeping difficulty | 5 (11·36) | 23 (22·12) | 0·127 | 1 (2·5) | 1 (3·7) | 0·776 |
| Lightening pain | 6 (13·64) | 7 (6·73) | 0·175 | 5 (12·5) | 0 (0·0) | 0·056 |
| Photophobia | 6 (13·64) | 8 (7·69) | 0·259 | 2 (5·0) | 1 (3·7) | 0·801 |
| No symptom | 6 (13·64) | 8 (7·69) | 0·259 | 5 (12·5) | 3 (11·1) | 0·863 |
| Cranial nerve impairs | 5 (11·36) | 7 (6·73) | 0·345 | 1 (2·5) | 2 (7·4) | 0·341 |
| Confusion | 2 (4·55) | 2 (1·92) | 0·369 | 2 (4·55) | 2 (1·92) | 0·369 |
| Blurred version | 4 (9·09) | 6 (5·77) | 0·462 | 3 (7·5) | 1 (3·7) | 0·520 |
| Myasthenia | 6 (13·64) | 11 (10·58) | 0·594 | 6 (13·64) | 11 (10·58) | 0·594 |
| Tremor | 2 (4·55) | 6 (5·77) | 0·763 | 2 (5·0) | 2 (7·4) | 0·683 |
| **Comorbidity** |  |  |  |  |  |  |
| Hypertension | 5 (11·36) | 9 (8·65) | 0·607 | 1 (2·5) | 0 (0·0) | 0·408 |
| Cardiovascular disease | 1 (2·27) | 2 (1·92) | 0·890 | 1 (2·5) | 2 (7·4) | 0·341 |
| Diabetes | 1 (2·27) | 4 (3·85) | 0·628 | 0 (0·0) | 0 (0·0) | - |
| Stroke | 1 (2·27) | 4 (3·85) | 0·628 | 2 (5·0) | 2 (7·4) | 0·683 |
| Chronic liver disease | 2 (4·55) | 4 (3·85) | 0·844 | 0 (0·0) | 0 (0·0) | NA |
| Dermatosis | 3 (6·82) | 4 (3·85) | 0·436 | 2 (5·0) | 1 (3·7) | 0·801 |
| Drug abuse | 1 (2·27) | 3 (2·88) | 0·834 | 0 (0·0) | 0 (0·0) | - |

NS: neurosyhilis

**Supplement 2 cost of neurosyphilis diagnosis tests**

| Items, price  (RMB / time) | Serum TRUST  49 | Serum TPPA  30 | Spinal tap operation* 200 | CSF Routine tests*  7 | CSF VDRL  135 | CSF  TRUST  49 | CSF TPPA  30 | Total  Price (RMB) |
| --- | --- | --- | --- | --- | --- | --- | --- | --- |
| Gold Standard Diagnosis Procedure | YES | YES | YES | YES | YES | YES | Optional alteration of VDRL | At least 470 |
| model | YES | YES | -- | -- | -- | -- | -- | 79 |

*Not including additional cost of admission of hospitalization

* CSF Routine: Protein, Glucose, Nucleated cells
